# There’s more to the picture than meets the eye: Reply to: Letter to the editor of Acta Neurochirurgica: Blind men and the elephant—comment on “The dentato-rubro-thalamic tract as the potential common deep brain stimulation target for tremor of various origin: an observational case series”

**DOI:** 10.1007/s00701-020-04348-z

**Published:** 2020-04-26

**Authors:** Volker A. Coenen, Bastian E. Sajonz, Marco Reisert, Horst Urbach, Peter C. Reinacher

**Affiliations:** 1 Department of Stereotactic and Functional Neurosurgery, Medical Faculty of Freiburg University, Freiburg University Medical Center, Freiburg i.Br., Germany; 2 Center for Deep Brain Stimulation, Freiburg University Medical Center, Freiburg i.Br., Germany; 3 Department of Neuroradiology, Medical Faculty of Freiburg University, Freiburg University Medical Center, Freiburg i.Br., Germany

We would like to thank *Drs. Low and Turner* for their thoughtful comments on our paper [1] and their friendly evaluation. We had indeed ourselves early formulated the theory that one fiber tract (DRT(T)) would possibly connect all stereotactic targets for tremor [2–4] (“three stereotactic targets, one anatomical (fiber) structure”). Other authors have looked at this matter and came to slightly different conclusions [5], although—especially for essential tremor—there is a growing literature successfully reporting DRT(T) targeting [6–9]. However, we should not forget that the subthalamic nucleus (STN) itself has clear tremor-reducing potency in Parkinson’s tremor [10] without a direct involvement of the DRTT and might therefore be an exception to this rule. We have in our workflow implemented DTI guided DRT(T) targeting for most tremor surgery (including Parkinson’s disease tremor) since 2011. However, the success of using the DTI technology for targeting depends on good individual imaging, robust reproduction of structures, and flawless tracking strategies. Moreover, neurosurgeons need to be aware of the actual accuracy of this technology which depends on post-processing of imaging and therefore has a certain amount of error [11]. This is especially important with respect to the beginning use of DRT(T) targeting in focused ultrasound lesioning procedures [12]. Low and Turner scientifically compared conventional versus DTI-based targeting strategies in their study [13], which is a logical approach [14]. We should—despite our enthusiasm–be aware of the slight possibility that the tractographically depicted DRT(T) might just be a surrogate for a yet to be discovered tremor-reducing anatomical structure which at this moment is invisible to our imaging technologies.
